# Relaxed imprinting of IGF2 in peripheral blood cells of patients with a history of prostate cancer

**DOI:** 10.1530/EC-12-0054

**Published:** 2012-10-24

**Authors:** Djeda Belharazem, Matthias Kirchner, Franziska Geissler, Peter Bugert, Martin Spahn, Burkhard Kneitz, Hubertus Riedmiller, Christian Sauer, Stefan Küffer, Lutz Trojan, Christian Bolenz, Maurice Stephan Michel, Alexander Marx, Philipp Ströbel

**Affiliations:** 1 Institute of Pathology, University Medical Center Mannheim, University of Heidelberg Theodor-Kutzer-Ufer 1–368135, Mannheim Germany; 2 Institute of Pathology Nordhessen Kassel Germany; 3 Institute of Transfusion Medicine and Immunology, University Medical Center Mannheim, University of Heidelberg Mannheim Germany; 4 Departments of Urology and Pediatric Urology University Hospital Würzburg Würzburg Germany; 5 University Medical Center Mannheim Mannheim Germany; 6 Institute of Pathology, University Medical Center Göttingen, University of Göttingen GöttingenGermany

**Keywords:** cancer, insulin-like growth factor 2, imprinting, prostate, screening

## Abstract

**Background:**

Insulin-like growth factor 2 (IGF2) is the predominant IGF in adults and regulates cell growth. In contrast to normal tissues, where IGF2 is imprinted and only expressed from the paternal allele, loss of imprinting (LOI) and biallelic IGF2 expression are observed in many cancers including prostate cancer (PCa). We here studied whether LOI of IGF2 in normal circulating peripheral blood lymphocytes can predict increased PCa risk.

**Samples and methods:**

We analyzed IGF2 protein levels, *IGF2 820G/A* genotype and imprinting status, as well as methylation status of the IGF2 imprinting control region (ICR) in 113 blood samples of patients with a history of radical prostatectomy (RPE) for PCa by ELISA, restriction-fragment length polymorphism, and bisulfite-DNA sequencing. Results were compared to 249 male blood donors with unknown prostate specific antigen (PSA) status.

**Results:**

The *820G/A* genotype was enriched in the RPE group and was associated with younger age at cancer diagnosis. LOI in patients was only slightly more frequent than in controls, but IGF2 levels were significantly higher and uncoupled from the imprinting status. Analysis of the *IGF2/H19* ICR revealed marked hypermethylation.

**Conclusions:**

The *IGF 820G/A* genotype is associated with PCa diagnosis at younger age. Increased IGF2 in patients with PCa appears to be the result of impaired imprinting in non-neoplastic cells rather than a paracrine tumor product. Uncoupling of IGF2 protein levels from imprinting status (not LOI alone) and hypermethylation of the ICR characterized PCa patients and could have the potential to indicate persons at risk in screening programs.

## Introduction

Prostate cancer (PCa) is the sixth most common cancer worldwide and the most prevalent cancer in men (1) with a lifetime risk of 16.48% (2). Older age is an important PCa risk factor, as about three-quarters of all cases occur in men over 65 years (3). Age dependence in cancers could be the result of accumulation of DNA damage (4) or epigenetic changes (5) in somatic cells. Imprinting is one mechanism of epigenetic gene regulation. Imprinted genes carry epigenetic marks (e.g. methylation) that usually allow expression of only one allele in a parent-of-origin-dependent fashion (6). Loss of imprinting (LOI) is considered one of the earliest (7) and most abundant alterations in cancer (8). Among the 90 imprinted genes known to date, the insulin-like growth factor 2 (*IGF2*) and *H19*, a gene for a noncoding RNA, are probably studied best (9). *IGF2* is of special interest, as it belongs to a small set of imprinted genes that can be studied in peripheral blood, shows monoallelic expression in normal blood lymphocytes, and has been linked to tumorigenesis (10). As epigenetic changes such as LOI and demethylation occur early in cancer progression, detection of such changes may be relevant for early cancer detection and prevention. LOI of *IGF2* has previously been described in normal circulating peripheral blood lymphocytes of individuals with an increased risk to develop colorectal cancer (11, 12, 13).

**Figure 1:**
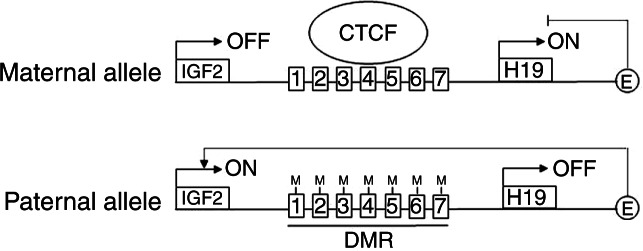
Schematic representation of the *IGF2/H19* locus. According to the CTCF boundary model, CTCF binds to unmethylated motifs within the seven imprinting control regions (ICRs) on the maternal allele and prevents activation of *IGF2* through blockade of enhancer elements (E). On the paternal allele, the ICRs are methylated, thereby preventing CTCF binding and leading to activation of *IGF2* through the enhancer.

*IGF2* and *H19* belong to the same locus and share similar expression patterns throughout most normal tissues. Imprinting of the *IGF2/H19* locus involves a so-called differentially methylated region (DMR) that acts as a boundary or insulator element. On the maternally inherited allele, the DMR is unmethylated allowing binding of the zinc finger protein CTCF to seven so-called imprinting control regions (ICRs) within the DMR, thereby preventing downstream enhancer elements from activating *IGF2*. On the paternally inherited allele, CTCF binding to the ICRs is blocked through methylation, allowing the downstream enhancers to interact with the *IGF2* promoter and expression of *IGF2* (14, 15) (Fig. 1). Using chromatin conformation capture, it was shown that these steps involve chromosomal looping and are accompanied by spatial translocation of the gene locus within the nucleus (16).

IGFs regulate cell growth and differentiation in humans. IGF2 is the predominant IGF in adults (17) and is imprinted in most tissues with few exceptions such as choroid plexus, leptomeninges, developing retina, and thymus (18, 19). LOI and increased expression of *IGF2* are observed in many cancers (20) including PCa (21, 22). Among other effects, biallelic IGF2 supply has been shown to enhance the effect of PTEN loss in transgenic animals (23).

To investigate whether LOI of *IGF2* was related also to the risk to develop PCa, we analyzed normal peripheral blood mononuclear cells (PBMNCs) from 113 male patients with a history of radical prostatectomy (RPE) for histologically proven PCa who were PSA negative at the time the blood sample was collected. Data were compared with blood samples from 246 PSA-negative healthy blood donors.

## Materials and methods

### Patient and control blood samples

Peripheral blood samples from 113 prostatectomized patients with histologically proven PCa (RPE patients) were collected 12–36 months after RPE (age range 47–85 years, median 67 years). Only persons with PSA levels ≤0.2 and without clinical evidence of disease at follow-up were included. As controls, we used 246 blood samples from volunteer blood donors (age range 19–82 years, median 43 years). Samples were not tested for PSA levels. Informed written consent was obtained from all tested individuals after full explanation of the purpose and nature of all procedures used. The study was approved by the Local Ethics Committee (ref no. Wü 59/04), functioning according to the 3rd edition of the Guidelines on the Practice of Ethical Committees in Medical Research issued by the Royal College of Physicians of London.

### Isolation of serum and nucleic acids from peripheral blood samples

Blood samples were processed within 1 h. Serum and PBMNCs were obtained after Ficoll–Hypaque density-gradient centrifugation as described (24). DNA and RNA were isolated from PBMNCs using the QIAamp kit (Qiagen) and the peqGold Blood RNA Kit (PeqLab, Erlangen, Germany) according to the manufacturer's instructions.

### ELISA

ELISA assays to detect serum IGF2 and IGFBP3 were purchased from Diagnostic System Laboratories (Webster, TX, USA) and performed according to the manufacturer's instructions. All serum samples were assayed in triplicates. The mean of the three values was used for further statistical evaluation.

### Single nucleotide polymorphism genotyping by restriction-fragment length polymorphism

Heterozygosity at the ApaI-sensitive 820G>A locus on exon 7 of the *IGF2* gene (GenBank sequence: X07868) was determined as described previously (25). In brief, DNA was amplified using primers F: 5′-CTTGGACTTTGAGTCAAATTGG-3′ and R: 5′-GGTCGTGCCAATTACATTTCA-3′, followed by HinfI and ApaI digestion.

### Analysis for LOI of IGF2

LOI or retention of imprinting (ROI) was determined by cDNA amplification from heterozygous cases by a nested RT-PCR method. In brief, total RNA was treated with DNAseI before RT. cDNA (1 μl) was amplified by 40 cycles of PCR using F: 5′-TCCTGGAGACGTACTGTGCTA-3′ and R: 5′-CGGGGATGCATAAAGTATGAG-3′ as primers. One microliter of the amplification product was used as a template for another 40 cycles using primers F: 5′-ACCGTGCTTCCGGACAAC-3′ (exon spanning) and R: 5′-GGTCGTGCCAATTACATTTCA-3′. The obtained PCR products were ethanol precipitated before direct DNA bidirectional sequencing using an ABI BigDye Terminator V1.1 cycle sequencing kit (Applied Biosystems, Inc., Foster City, CA, USA) and analyzed on a Genetic Analyzer 3110 (Applied Biosystems). The sequencing results were analyzed with CHROMAS Software version 2.0 (Technelysium Pty Ltd., South Brisbane, Australia). LOI was calculated as the ratio of the less active allele/more active allele, with LOI defined as an LOI index >0.25. With this definition, the mean LOI index of cases with intact imprinting was 0.16±0.05 and was not different between controls and RPE patients. The mean LOI index of cases with LOI was 0.55±0.22 and was also not different between controls and RPE patients (*P*=0.06).

### Bisulfite treatment

DNA methylation analyses, starting with 1–2 μg DNA, were carried out using bisulfite treatment and subsequent sequencing as described previously (26). For PCR amplification of bisulfite-treated DNA, primers specific for methylated and unmethylated sequences spanning the seven CpG-rich *IGF2* ICRs located 2 kb upstream of the *H19* transcription site were designed after published sequences (GenBank ID: 283120; primers available upon request). The obtained PCR products were sequenced and the results were analyzed using CHROMAS Software version 2.0. The relative percentage of methylated vs unmethylated bases at a given CG site was calculated by dividing the amplitudes for C's (i.e. methylated bases) by the amplitudes for T's (i.e. unmethylated bases).

### Statistical analyses

The allelic frequencies were compared with *χ* ^2^-test calculated on 2×2 contingency tables. The odds ratio (OR) was calculated to demonstrate the strength of difference between groups of subjects. *χ* ^2^-Test was also used to test for deviation from Hardy–Weinberg equilibrium. Mann–Whitney *U* test was used for statistical comparisons between unrelated groups. Spearman's test was used to test for correlations between variables. A *P* value <0.05 was considered statistically significant.

## Results

### Biased distribution of 820G/A alleles in prostate carcinoma patients

**Figure 2:**
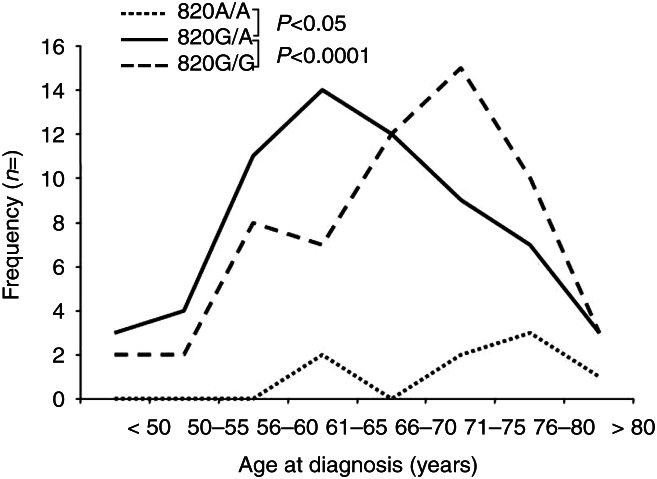
Correlation between IGF2 820G/A allele status and age at tumor diagnosis in patients with a history of prostate cancer. Patients with an 820G/A genotype were on average 4 years younger than patients with an 820G/G genotype and 8 years younger than patients with an 820A/A genotype.

The distribution of the 820G/A genotypes in RPE patients and control persons is summarized in Table 1. Among men with a history of PCa, the 820G/A genotype was significantly more frequent than among healthy control persons (50.5% in RPE patients vs 43% in controls; OR 1.92, 95% confidence interval 1.22–3.02, *P*=0.005), resulting in a deviation from expected Hardy–Weinberg proportions (*P*=0.001). Of note, among RPE patients, men with 820G/A were significantly younger (median age at diagnosis 65 years, range 49–83) at PCa diagnosis than men with the G/G (median age at diagnosis 69 years, range 47–85) or the A/A genotype (median age at diagnosis 73 years, range 65–85) (*P*<0.0001; Fig. 2). 820G/A was previously found to be associated with body mass index (BMI) (27). To test whether the observed age difference were linked to differences in body weight, we compared BMI of 820G/A patients to those with other genotypes but found no statistical difference (*P*=0.17, data not shown).

### LOI and increased IGF2 levels in the serum of PSA-negative RPE patients

**Figure 3:**
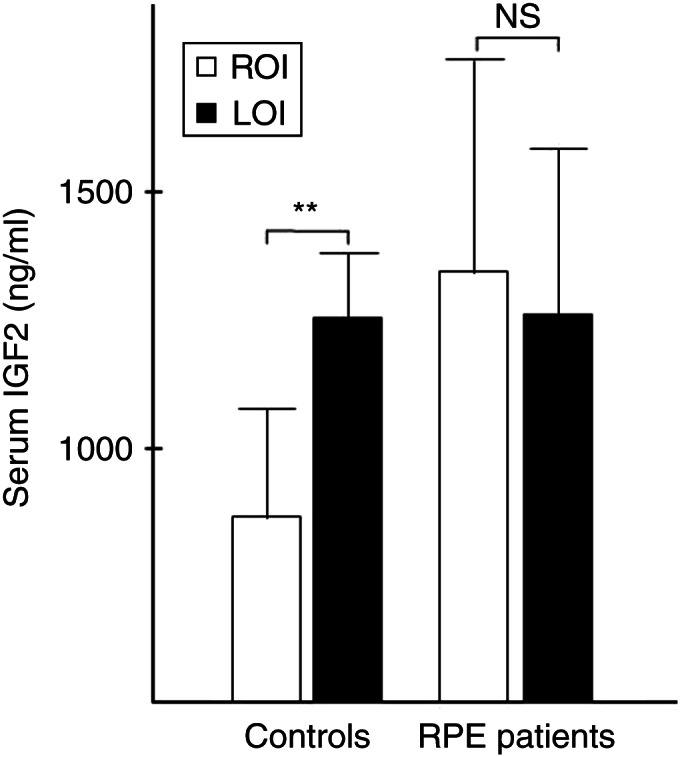
Circulating IGF2 protein levels in patients with a history of prostate cancer (RPE patients) and control persons. In controls, IGF2 levels were significantly higher in persons with loss of imprinting (LOI) compared with persons with ROI with an average increase of 35% in persons with LOI (**P=0.001). In RPE patients, in contrast, there was no difference between cases with and without LOI (NS, not significant).

cDNA from 106 informative cases (41 RPE patients and 65 controls with 820G/A genotype) was tested for LOI status. In control persons, IGF2 levels were tightly correlated with *IGF2* imprinting status (*r*=0.6, *P*<0.05). LOI was present in 13/65 (20%) of the samples and was associated with a 35% increase in IGF2 levels in the serum (IGF2 in control persons with ROI 993±172.8 ng/ml vs LOI 1302±107.5 ng/ml, *P*=0.001; Fig. 3). The percentage of cases with LOI in control persons was identical for all age groups and did not increase with age.

LOI in RPE patients was significantly more frequent (16/41 cases (39%), *P*=0.03). In contrast to controls, IGF2 levels in all RPE patients were significantly increased and appeared uncoupled from the imprinting status (IGF2 levels in patients with ROI 1377±333.6 ng/ml vs LOI 1310±126.1 ng/ml, *P*=0.9; Fig. 3). Interestingly, circulating IGF2 levels in LOI cases in controls and ROI/LOI in RPE patients were similar, suggesting that under normal conditions, ∼35% of circulating IGF2 in the serum is regulated by imprinting. ELISA testing showed that IGF2 protein levels both in patients and in controls were tightly correlated with IGFBP3 levels (*r*=0.8, *P*<0.0001; data not shown), indicating that the vast majority of the detected IGF2 was bound to IGFBP3.

### Hypermethylation of IGF2 ICRs in blood cells of RPE patients

**Figure 4:**
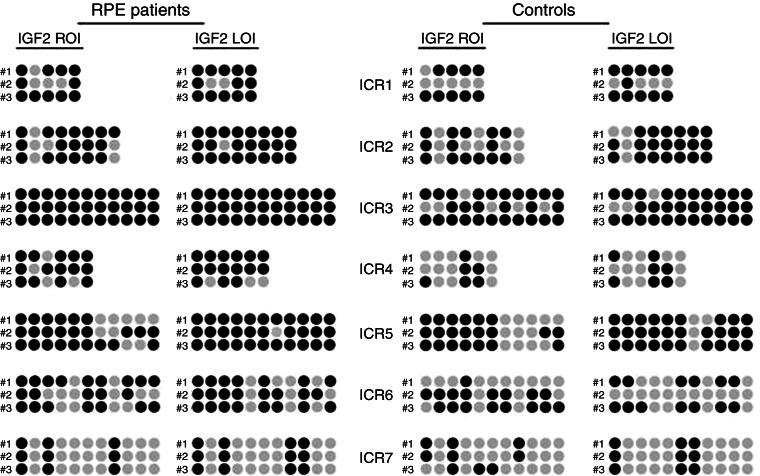
Comparison of imprinting status of the imprinting control regions (ICR) 1–7 within the *IGF2/H19* locus in six patients with a history of prostate cancer (RPE) and six controls (schematic representation of results: black circles, methylated; gray circles, unmethylated CGs). In both groups, three cases (nos 1–3) with either loss (LOI) or retention (ROI) of *IGF2* imprinting were analyzed. In controls with LOI, all ICRs were hypermethylated compared to cases with ROI. In RPE patients, ICRs showed a higher degree of methylation. Note complete methylation of CGs within ICR3.

To further investigate the reasons for the observed uncoupling of *IGF2* levels from imprinting status in RPE patients, we analyzed methylation of the ICRs 1–7 (Fig. 4). Hypermethylation in one or several of these regions is thought to interfere with CTCF binding, to favor loss of *IGF2* imprinting and hence to increase *IGF2* expression. In general agreement with the CTCF boundary model, we observed a higher degree of methylation in samples with LOI than in ROI in both RPE patients (LOI vs ROI, 36–100% methylation; mean, 80.9 vs 28–100% methylation; mean value, 68.7%) and controls (LOI vs ROI, 27–91% methylation; mean, 62.7 vs 27–82% methylation; mean, 53.7%). However, all ICRs in samples of RPE patients, irrespective of the imprinting status, showed a higher degree of methylation compared with control samples (percent methylated bases RPE vs controls (ROI samples), ICR1 63 vs 60%; ICR2 75 vs 58%; ICR3 100 vs 82%; ICR4 78 vs 33%; ICR5 73 vs 64%; ICR6 64 vs 52%; and ICR7 28 vs 27%). We hypothesize that IGF2 imprinting in peripheral blood cells of RPE patients may be incomplete due to global ICR hypermethylation.

## Discussion

It has previously been reported that LOI for *IGF2* is an epigenetic marker of colorectal cancer risk (12). In a similar approach, we studied IGF2 and its regulation in the peripheral blood of patients with a history of PCa who where PSA negative at the time when the blood sample was collected. It is a commonly held view that increased circulating IGF2 is a tumor product (28, 29). We here show that this traditional view may be inaccurate for PCa patients: our findings in non-neoplastic blood cells rather point to a general relaxation of *IGF2* imprinting in these patients. We found enrichment of the 820G/A genotype among patients with a history of PCa (OR 1.92, *P*=0.005), a finding that may merit further consideration in future population studies. However, this retrospective study was not intended nor statistically powered to detect risk alleles in a general population. Thus, the more important finding was the fact that 820G/A genotype was a risk factor among RPE patients, as men with 820G/A were on average 5 years younger at PCa diagnosis than patients with a *G/G* or *A/A* genotype. The reasons for this observation are unclear. 820G/A was previously found to be associated with BMI (27, 30). Currently available data suggest a possible correlation between obesity and PCa risk (31, 32, 33). However, we found no correlation between 820G/A alleles and BMI (data not shown). *IGF2* lies in a 19 kb genomic PCa risk region on chromosome 11p15.5, together with insulin (INS) and tyrosine hydroxylase (TH) (34). Many of the single nucleotide polymorphisms (SNPs) in this region are in tight linkage disequilibrium with each other. In line with previous reports, we did not observe co-segregation of 820G/A with other published risk alleles (+1127 *INS*-PstI and −4217 *TH*-PstI) (35) (data not shown). This strongly suggests that 820G/A is a putative risk allele of its own.

Using the 820G/A SNP to study imprinting of IGF2, we found that 20% of healthy male control persons showed LOI. In line with previous observations (11, 12), the percentage of LOI cases was identical in all age groups, suggesting that LOI in peripheral blood cells is a genetically imposed condition rather than an age-related phenomenon. Cui *et al*. (12) found LOI in peripheral blood lymphocytes to be predictive of LOI in both normal and cancerous colonic mucosa of patients with colorectal cancer. Interestingly, Fu *et al*. (22) reported a more pronounced relaxation of imprinting in prostates of men with PCa than in age-matched control patients without PCa. These previous observations together with the data presented here raise the possibility that LOI in peripheral blood cells reflects a general impairment of imprinting rather than a tissue-restricted pre-neoplastic process. LOI in control persons resulted in a 35% increase in circulating IGF2. In stark contrast to control persons, in whom imprinting status and *IGF2* expression level were tightly correlated, *IGF2* imprinting and expression were uncoupled in RPE patients and IGF2 protein levels were also increased (again by 35%) in patients with ROI. These results indicate that ∼35% of circulating IGF2 is controlled by imprinting. One possible explanation for the increased serum IGF2 came from the observation that the ICRs 1–7 in peripheral blood cells of RPE patients were moderately hypermethylated, suggesting impaired CTCF binding and consequent incomplete or ‘leaky’ imprinting. Discordance between methylation and imprinting status at the *IGF2/H19* locus has been previously observed (36) pointing to epigenetic mechanisms other than DNA methylation (10). Importantly, increased IGF2 protein levels in spite of apparently retained imprinting at the *IGF2/H19* locus were observed not only in virtually 100% RPE patients with ROI but also in a few control persons. As the controls of this study were persons who were anonymized blood donor volunteers that had not been interviewed for a history of prostatic disease nor tested for PSA levels, this raises the possibility that those few control persons with uncoupling of IGF2 from imprinting may have had occult PCa.

In conclusion, our results suggest that assessment of the *IGF2 820G/A* locus may provide clinically relevant information with respect to PCa risk and age of onset. Uncoupling of IGF2 imprinting from IGF2 levels and hypermethylation of the ICR in circulating peripheral blood lymphocytes may indicate persons at risk warranting increased surveillance. It will be important to test this prediction in a longitudinal prospective cohort in persons at risk to develop PCa and eventually also other cancers.

## Author contribution statement

D Belharazem, M Kirchner, F Geissler, P Bugert, C Sauer, B Kneitz, and S Küffer performed experiments and assisted in the preparation of the manuscript. P Bugert, M Spahn, H Riedmiller, L Trojan, C Bolenz, and M S Michel were in charge of patients, collected patient and control samples, and participated in writing of the manuscript. A Marx and P Ströbel were principal investigators who designed and supervised the study and were involved in writing and editing the manuscript.

## Figures and Tables

**Table 1:** 820G/A (ApaI) genotype frequencies and Hardy–Weinberg (HW) equilibrium test in peripheral blood mononuclear cells (PBMNCs) in the blood of patients with a history of radical prostatectomy (RPE) for prostate cancer.

	***n***	**G/G**	**A/G**	**A/A**	***P* value for HW**
PBMNC RPE	113	40 (43%)	67 (50.5%)	6 (6.5%)	0.001
PBMNC controls	246	119 (48.5%)	106 (43%)	21 (8.5%)	0.702

The allelic frequencies between RPE patients and controls were statistically different (*P*=0.02).

## References

[1] Parkin DM (2001). Global cancer statistics in the year 2000. Lancet Oncology.

[2] Howlader N, Noone AM, Krapcho M, Neyman N, Aminou R, Altekruse SF, Kosary CL, Ruhl J, Tatalovich Z, Cho H, Mariotto A, Eisner MP, Lewis DR, Chen HS, Feuer EJ, Cronin KA (eds). SEER Cancer Statistics Review, 1975-2009 (Vintage 2009 Populations), National Cancer Institute. Bethesda, MD, http://seer.cancer.gov/csr/1975_2009_pops09/, based on November 2011 SEER data submission, posted to the SEER web site, April 2012.

[3] Epstein JI, Algaba F, Allsbrook Jr WC, Bastacky S, Boccon-Gibod L, De Marzo AM, Egevad L, Furusato M, Hamper UM, Helpap B, Humphrey PA, Iczkowski KA, Lopez-Beltran A, Montironi R, Rubin MA, Sakr WA, Samaratunga H & Parkin DM. Tumours of the prostate. In *World Health Organization Classification of Tumours Pathology and Genetics of Tumours of the Urinary System and Male Genital Organs*, p 163. Eds JN Eble, G Sauter, JI Epstein & IA Sesterhenn. Lyon: IARC Press, 2004.

[4] Malins DC, Johnson PM, Barker EA, Polissar NL, Wheeler TM, Anderson KM (2003). Cancer-related changes in prostate DNA as men age and early identification of metastasis in primary prostate tumors. PNAS.

[5] Kwabi-Addo B, Chung W, Shen L, Ittmann M, Wheeler T, Jelinek J, Issa JP (2007). Age-related DNA methylation changes in normal human prostate tissues. Clinical Cancer Research.

[6] Bartolomei MS (2009). Genomic imprinting: employing and avoiding epigenetic processes. Genes and Development.

[7] Jelinic P, Shaw P (2007). Loss of imprinting and cancer. Journal of Pathology.

[8] Feinberg AP, Ohlsson R, Henikoff S (2006). The epigenetic progenitor origin of human cancer. Nature Reviews Genetics.

[9] Barlow DP, Stoger R, Herrmann BG, Saito K, Schweifer N (1991). The mouse insulin-like growth factor type-2 receptor is imprinted and closely linked to the Tme locus. Nature.

[10] Frost JM, Monk D, Stojilkovic-Mikic T, Woodfine K, Chitty LS, Murrell A, Stanier P, Moore GE (2010). Evaluation of allelic expression of imprinted genes in adult human blood. PLoS ONE.

[11] Cruz-Correa M, Zhao R, Oviedo M, Bernabe RD, Lacourt M, Cardona A, Lopez-Enriquez R, Wexner S, Cuffari C, Hylind L, Platz E, Cui H, Feinberg AP, Giardiello FM (2009). Temporal stability and age-related prevalence of loss of imprinting of the insulin-like growth factor-2 gene. Epigenetics.

[12] Cui H, Cruz-Correa M, Giardiello FM, Hutcheon DF, Kafonek DR, Brandenburg S, Wu Y, He X, Powe NR, Feinberg AP (2003). Loss of IGF2 imprinting: a potential marker of colorectal cancer risk. Science.

[13] Kaneda A, Feinberg AP (2005). Loss of imprinting of IGF2: a common epigenetic modifier of intestinal tumor risk. Cancer Research.

[14] Hark AT, Schoenherr CJ, Katz DJ, Ingram RS, Levorse JM, Tilghman SM (2000). CTCF mediates methylation-sensitive enhancer-blocking activity at the H19/Igf2 locus. Nature.

[15] Kanduri C, Pant V, Loukinov D, Pugacheva E, Qi CF, Wolffe A, Ohlsson R, Lobanenkov VV (2000). Functional association of CTCF with the insulator upstream of the H19 gene is parent of origin-specific and methylation-sensitive. Current Biology.

[16] Murrell A, Heeson S, Reik W (2004). Interaction between differentially methylated regions partitions the imprinted genes Igf2 and H19 into parent-specific chromatin loops. Nature Genetics.

[17] LeRoith D, Roberts CT (2003). The insulin-like growth factor system and cancer. Cancer Letters.

[18] Ohlsson R, Hedborg F, Holmgren L, Walsh C, Ekstrom TJ (1994). Overlapping patterns of IGF2 and H19 expression during human development: biallelic IGF2 expression correlates with a lack of H19 expression. Development.

[19] Derbinski J, Gabler J, Brors B, Tierling S, Jonnakuty S, Hergenhahn M, Peltonen L, Walter J, Kyewski B (2005). Promiscuous gene expression in thymic epithelial cells is regulated at multiple levels. Journal of Experimental Medicine.

[20] Reik W, Constancia M, Dean W, Davies K, Bowden L, Murrell A, Feil R, Walter J, Kelsey G (2000). Igf2 imprinting in development and disease. International Journal of Developmental Biology.

[21] Jarrard DF, Bussemakers MJ, Bova GS, Isaacs WB (1995). Regional loss of imprinting of the insulin-like growth factor II gene occurs in human prostate tissues. Clinical Cancer Research.

[22] Fu VX, Dobosy JR, Desotelle JA, Almassi N, Ewald JA, Srinivasan R, Berres M, Svaren J, Weindruch R, Jarrard DF (2008). Aging and cancer-related loss of insulin-like growth factor 2 imprinting in the mouse and human prostate. Cancer Research.

[23] Church DN, Phillips BR, Stuckey DJ, Barnes DJ, Buffa FM, Manek S, Clarke K, Harris AL, Carter EJ, Hassan AB (2012). Igf2 ligand dependency of Pten(+/−) developmental and tumour phenotypes in the mouse. Oncogene.

[24] Nenninger R, Schultz A, Hoffacker V, Helmreich M, Wilisch A, Vandekerckhove B, Hunig T, Schalke B, Schneider C, Tzartos S, Kalbacher H, Muller-Hermelink HK, Marx A (1998). Abnormal thymocyte development and generation of autoreactive T cells in mixed and cortical thymomas. Laboratory Investigation.

[25] Ogawa O, Eccles MR, Szeto J, McNoe LA, Yun K, Maw MA, Smith PJ, Reeve AE (1993). Relaxation of insulin-like growth factor II gene imprinting implicated in Wilms' tumour. Nature.

[26] Strobel P, Chuang WY, Chuvpilo S, Zettl A, Katzenberger T, Kalbacher H, Rieckmann P, Nix W, Schalke B, Gold R, Muller-Hermelink HK, Peterson P, Marx A (2008). Common cellular and diverse genetic basis of thymoma-associated myasthenia gravis: role of MHC class II and AIRE genes and genetic polymorphisms. Annals of the New York Academy of Sciences.

[27] Gaunt TR, Cooper JA, Miller GJ, Day IN, O'Dell SD (2001). Positive associations between single nucleotide polymorphisms in the IGF2 gene region and body mass index in adult males. Human Molecular Genetics.

[28] Heude B, Ong KK, Luben R, Wareham NJ, Sandhu MS (2007). Study of association between common variation in the insulin-like growth factor 2 gene and indices of obesity and body size in middle-aged men and women. Journal of Clinical Endocrinology and Metabolism.

[29] Trojan L, Bode C, Weiss C, Mayer D, Grobholz R, Alken P, Michel MS (2006). IGF-II serum levels increase discrimination between benign prostatic hyperplasia and prostate cancer and improve the predictive value of PSA in clinical staging. European Urology.

[30] O'Dell SD, Miller GJ, Cooper JA, Hindmarsh PC, Pringle PJ, Ford H, Humphries SE, Day IN (1997). Apal polymorphism in insulin-like growth factor II (IGF2) gene and weight in middle-aged males. International Journal of Obesity and Related Metabolic Disorders.

[31] Discacciati A, Orsini N, Andersson SO, Andren O, Johansson JE, Wolk A (2011). Body mass index in early and middle-late adulthood and risk of localised, advanced and fatal prostate cancer: a population-based prospective study. British Journal of Cancer.

[32] Discacciati A, Orsini N, Wolk A (2012). Body mass index and incidence of localized and advanced prostate cancer – a dose–response meta-analysis of prospective studies. Annals of Oncology.

[33] Guh DP, Zhang W, Bansback N, Amarsi Z, Birmingham CL, Anis AH (2009). The incidence of co-morbidities related to obesity and overweight: a systematic review and meta-analysis. BMC Public Health.

[34] Eeles RA, Kote-Jarai Z, Al Olama AA, Giles GG, Guy M, Severi G, Muir K, Hopper JL, Henderson BE, Haiman CA, Schleutker J, Hamdy FC, Neal DE, Donovan JL, Stanford JL, Ostrander EA, Ingles SA, John EM, Thibodeau SN, Schaid D, Park JY, Spurdle A, Clements J, Dickinson JL, Maier C, Vogel W, Dork T, Rebbeck TR, Cooney KA, Cannon-Albright L, Chappuis PO, Hutter P, Zeegers M, Kaneva R, Zhang HW, Lu YJ, Foulkes WD, English DR, Leongamornlert DA, Tymrakiewicz M, Morrison J, Ardern-Jones AT, Hall AL, O'Brien LT, Wilkinson RA, Saunders EJ, Page EC, Sawyer EJ, Edwards SM, Dearnaley DP, Horwich A, Huddart RA, Khoo VS, Parker CC, Van As N, Woodhouse CJ, Thompson A, Christmas T, Ogden C, Cooper CS, Southey MC, Lophatananon A, Liu JF, Kolonel LN, Le Marchand L, Wahlfors T, Tammela TL, Auvinen A, Lewis SJ, Cox A, FitzGerald LM, Koopmeiners JS, Karyadi DM, Kwon EM, Stern MC, Corral R, Joshi AD, Shahabi A, McDonnell SK, Sellers TA, Pow-Sang J, Chambers S, Aitken J, Gardiner RA, Batra J, Kedda MA, Lose F, Polanowski A, Patterson B, Serth J, Meyer A, Luedeke M, Stefflova K, Ray AM, Lange EM, Farnham J, Khan H, Slavov C, Mitkova A, Cao G, Easton DF (2009). Identification of seven new prostate cancer susceptibility loci through a genome-wide association study. Nature Genetics.

[35] Ho GY, Melman A, Liu SM, Li M, Yu H, Negassa A, Burk RD, Hsing AW, Ghavamian R, Chua SC (2003). Polymorphism of the insulin gene is associated with increased prostate cancer risk. British Journal of Cancer.

[36] Ulaner GA, Yang Y, Hu JF, Li T, Vu TH, Hoffman AR (2003). CTCF binding at the insulin-like growth factor-II (IGF2)/H19 imprinting control region is insufficient to regulate IGF2/H19 expression in human tissues. Endocrinology.

